# Changes in Glutathione Content in Liver Diseases: An Update

**DOI:** 10.3390/antiox10030364

**Published:** 2021-02-28

**Authors:** Mariapia Vairetti, Laura Giuseppina Di Pasqua, Marta Cagna, Plinio Richelmi, Andrea Ferrigno, Clarissa Berardo

**Affiliations:** Unit of Cellular and Molecular Pharmacology and Toxicology, Department of Internal Medicine and Therapeutics, University of Pavia, 27100 Pavia, Italy; mariapia.vairetti@unipv.it (M.V.); marta.cagna02@universitadipavia.it (M.C.); plinio.richelmi@unipv.it (P.R.); clarissa.berardo01@universitadipavia.it (C.B.)

**Keywords:** glutathione, liver, Nrf2

## Abstract

Glutathione (GSH), a tripeptide particularly concentrated in the liver, is the most important thiol reducing agent involved in the modulation of redox processes. It has also been demonstrated that GSH cannot be considered only as a mere free radical scavenger but that it takes part in the network governing the choice between survival, necrosis and apoptosis as well as in altering the function of signal transduction and transcription factor molecules. The purpose of the present review is to provide an overview on the molecular biology of the GSH system; therefore, GSH synthesis, metabolism and regulation will be reviewed. The multiple GSH functions will be described, as well as the importance of GSH compartmentalization into distinct subcellular pools and inter-organ transfer. Furthermore, we will highlight the close relationship existing between GSH content and the pathogenesis of liver disease, such as non-alcoholic fatty liver disease (NAFLD), alcoholic liver disease (ALD), chronic cholestatic injury, ischemia/reperfusion damage, hepatitis C virus (HCV), hepatitis B virus (HBV) and hepatocellular carcinoma. Finally, the potential therapeutic benefits of GSH and GSH-related medications, will be described for each liver disorder taken into account.

## 1. Introduction

**Figure 1:**
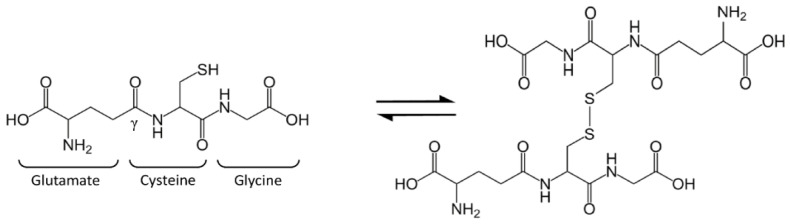
Glutathione (GSH) structure and balance between GSH and glutathione disulfide (GSSG).

Glutathione (GSH), or l-γ-glutamyl-l-cysteinyl-glycine, is a tripeptide present in mammalian cells at surprisingly high levels (1–10 mM) and particularly concentrated in the liver [1,2]. The thiol group of cysteine is responsible for GSH biological activity: GSH is considered the most important cellular redox buffer and major defender against oxidative stress. GSH is oxidized into glutathione disulfide (GSSG), which is actually two GSH molecules bound together at the sulfur atoms (Figure 1). The oxidized form is reconverted into GSH by the NADPH-dependent enzyme glutathione reductase (GR) [3]. The GSH:GSSG ratio is often used as a marker of cellular toxicity. Under normal conditions, the molar GSH:GSSG ratio is around 100:1; during stress, this ratio has been shown to decrease to 10:1 and even 1:1 [4,5,6,7,8].

It has been demonstrated that GSH has multiple roles in cell physiology, including:

(i) direct scavenging of reactive oxygen species (ROS), nitric oxide (NO) and its derivatives (RNS), resulting in the protection of the electron transport chain, DNA, lipids and proteins; (ii) indirect neutralization of intoxicants; (iii) S-gluta-thionylation of protein thiol groups; (iv) regulation of cell cycle progression and apoptosis.

From these considerations, it emerges that GSH cannot be considered a mere free radical scavenger but that it has a weighty role in the network governing the choice between survival, necrosis and apoptosis [9,10] as well as in cell signaling [10,11] and metabolism [3,12].

The present review will focus on the molecular biology of the GSH system. GSH synthesis, metabolism and regulation will be reviewed; the multiple GSH functions will be described, as well as GSH compartmentalization and interorgan transfer. Finally, the role of GSH in pathogenesis and potential therapeutic approaches will be also highlighted.

## 2. Glutathione Synthesis and Metabolism

The main source of GSH is the liver, which has the unique ability to synthesize cysteine, a GSH precursor, from endogenous sources through the trans-sulphuration pathway [13], or to obtain it from protein breakdown and food. GSH availability in hepatocytes depends on the membrane transport of cysteine itself, cystine and methionine [2]. In fact, a good amount of GSH is indirectly obtained from methionine; methionine deficiency has been found to induce a reduction in GSH availability, especially in the liver [14,15]. As a consequence, the liver plays a central role in maintaining interorgan homeostasis of GSH by exporting nearly all of the synthesized GSH into plasma and bile; in fact, the liver has the highest capacity for GSH efflux through its basolateral and apical poles [13]. Consequently, an alteration of the hepatic ability to synthesize or export GSH has an impact on systemic GSH homeostasis [2].

The first step in GSH synthesis is catalyzed by l-glutamate l-cysteine γ-ligase (GCL), resulting in the formation of γ-glutamyl-cysteine. GCL is a heterodimer constituted by a catalytic monomer (GCLC) and a modifier monomer (GCLM). GCLC is sensitive to GSH-induced feedback inhibition. GCLM is enzymatically inert; however, in the dimer it reduces the inhibitory feedback response to GSH [2]. GCL expression responds to oxidative stress and is positively regulated by nuclear factor erythroid 2-related factor 2 (Nrf2) [16,17,18,19]. Nrf2 works in combination with small avian musculoaponeurotic fibrosarcoma oncogene homolog (MAF) proteins: together they recognize and bind antioxidant response elements (ARE) in promoters of antioxidant enzyme genes (phase 2 enzymes such as glutathione S-Transferase (GST), UDP-glucorosyl transferase and superoxide dismutase (SOD), GSH peroxidase (GPx) and catalase (CAT), sulfiredoxin 1 and Thioredoxin reductase 1 [16,17,18,19]. Nrf2 is inhibited by Kelch-like ECH-associated protein 1 (Keap1): specifically, it increases Nrf2 proteasome-mediated degradation [20,21]. Electrophilic agents and antioxidants induce conformational changes in Keap1, thus impeding the binding with Nrf2 leading to the consequent migration of the nuclear factor in the nucleus, where it can exert its function [22].

The second step in GSH synthesis is catalyzed by GSH synthetase (GS), which produces GSH by adding a glycine molecule to γ-glutamyl-cysteine. Both first and second steps require energy in the form of adenosine triphosphate (ATP); however, ATP is not usually considered a limiting factor in GSH production except in pathological conditions [10]. The rate-limiting enzyme is considered to be GCL [2]; in fact, by overexpressing GCL and not GS, a net increase in GSH was obtained in *Saccharomyces cerevisiae* [23].

Usually, amino acids are linked together by an α-carboxyl group; differently, in GSH, the peptide bond linking glutamate and cysteine is constituted by a γ-carbonyl group, which is resistant to the hydrolytic action of cellular peptidases. Consequently, GSH is highly stable intracellularly [24] and its degradation occurs only extracellularly. γ-glutamyl transpeptidase (GGT), the enzyme responsible for GSH degradation, is located on the external surface of epithelial cells in kidney tubules, biliary epithelium and brain capillaries, allowing GSH amino acidic constituents to be recycled in the so-called γ-glutamyl cycle for intracellular GSH synthesis [25].

The utilization of GSH is consequential to its chemical structure. In particular, the thiol group of GSH undergoes a one-electron reduction with radicals, producing an unstable GS^•^. This reaction is kinetically driven by two further reactions: the first one with thiolate anion GS¯, and the second one with oxygen, producing GSSG and the superoxide anion O_2_^•^¯. The process is then completed by SOD and CAT, converting O_2_^•^¯ into H_2_O and O_2_ [10].

**Figure 2:**
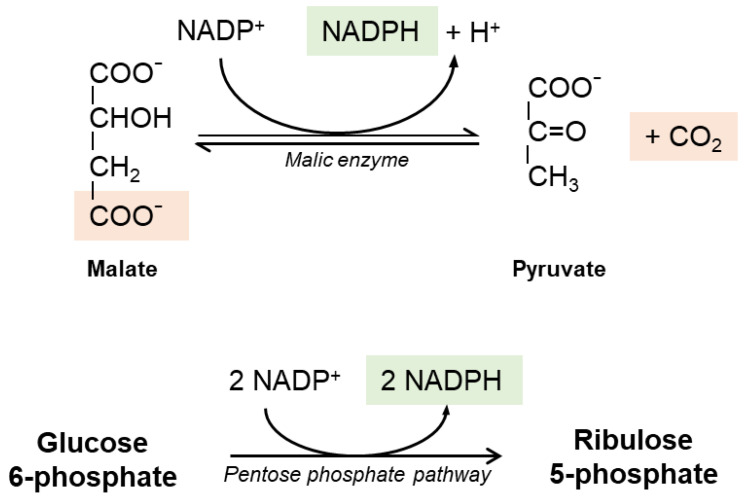
The pentose phosphate pathway and the malic enzyme reaction. The pentose phosphate pathway is considered the main source of reducing equivalents used for GSH regeneration; it is also the sole source of NADPH in red blood cells. The malic enzyme is another relevant source of NADPH in hepatic cells.

It is widely believed that the reductive power needed to recycle GSSG into GSH is provided by cellular Nicotinamide Adenine Dinucleotide Phosphate (NADPH) generated in the pentose phosphate pathway (Figure 2); the first step is catalyzed by glucose-6-phosphate dehydrogenase (G6PDH) and results in the oxidation of a glucose-6-phosphate into 6-phosphoglucolactone with the release of one NADPH. The second reaction of the cycle is catalyzed by 6-phosphogluconate dehydrogenase (6-PGDH). NADPH-dependent malic enzyme (ME)-1 also has a role in hepatic GSH production. Cytosolic ME-1 is a homo-tetrameric enzyme catalyzing reversible decarboxylation of malate into pyruvate and carbon dioxide; in the process, ME-1 generates NADPH. However, hepatocytes do not strictly depend from ME-1 activity for NADPH synthesis: in ME-1 deficient mice, after an initial depletion, the GSH levels recover quickly and sensitivity to acetaminophen-induced liver injury was unaltered [26].

## 3. Glutathione Functions

### 3.1. Direct Antioxidant Activity

Normally, the ROS-generating processes are counterbalanced by the antioxidant system, in which GSH is considered a key player. However, under pathological conditions, ROS level increases above the steady-state leading to oxidative stress, defined as a situation where the steady-state ROS concentration is transiently or chronically enhanced, disturbing cellular metabolism and its regulation and damaging cellular constituents [3]. When the cellular antioxidant defense system is overwhelmed by ROS production, the imbalance can culminate in cell death by apoptosis or necrosis [3]. In the process of counteracting the increase in oxidative stress, energy and amino acids are consumed resulting in the restoration of normal ROS level. Alternatively, in case of prolonged increase in ROS production, the instauration of a chronic condition may lead to various pathologies, such as cardiovascular diseases, chronic obstructive pulmonary disease, chronic kidney disease, neurodegenerative diseases, cancer and non-alcoholic steatohepatitis [27,28].

GSH directly scavenges ROS and RNS such as HO^•^, RO^•^, RO_2_^•^, HOCl, _1_O^2^ and ONOO¯. NO was originally believed to be directly scavenged by GSH to produce *S*-nitroso-glutathione (GSNO). However, it has been demonstrated that NO firstly needs to be converted into a nitrosium ion (NO^+^) before being able to react with GSH to form GSNO [3]. It should be noted that GSNO is not a terminal GSH metabolite; it should rather be considered as a GSH and NO storage and transportation system. For example, NO is released from GSNO when reacting with nitrosylating enzymes, such as thioredoxin and GSNO reductase; the three of them are capable of protein nitrosylation/denitrosylation, depending on their oxidation state [29].

### 3.2. Detoxification of Endogenous Compounds and Xenobiotics

GSH and GSH-related enzymes have a relevant role in cellular detoxification. GSH is a nucleophile and therefore it can react with electrophilic species transforming them into more soluble and easily excretable molecules. Conjugation of GSH with electrophilic compounds is mainly mediated by the GSTs, a super family of Phase II detoxification enzymes classified, according to their localization and properties, into cytosolic, mitochondrial and microsomal GSTs [30].

**Figure 3:**
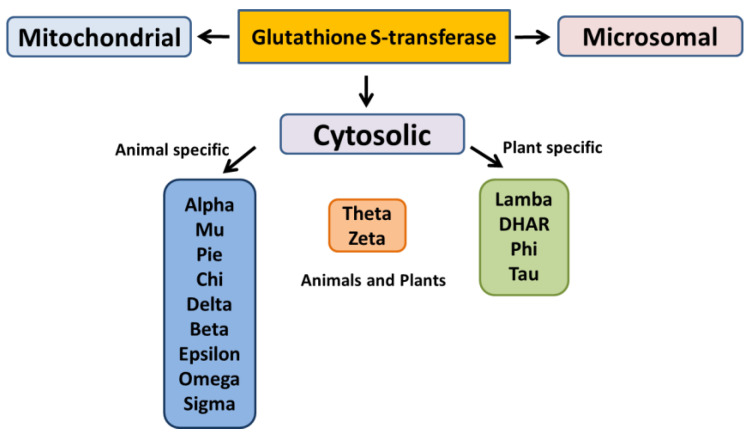
Different classes of glutathione S-transferase.

The cytosolic GSTs have been divided into different classes based on their genesis, catalytic amino-acid residue, sequence similarity, and substrate specificity [31]. These distinct evolutionary classes include Alpha, Beta, Dehydro-ascorbate reductases (DHARs), Delta, Epsilon, Mu, Omega, Pi, Phi, Sigma, Tau, Theta, and Zeta. Among these classes, only six have been functionally characterized in plants (Figure 3). Lambda class has structural similarity with Omega GSTs of mammals; both the classes share the number of conserved residues in the N-terminal GSH binding domain and possess a distinct structural feature of N-terminal extension [32].

The microsomal GSTs have been identified as the integral membrane proteins and are known as Membrane-Associated Proteins in Eicosanoid and Glutathione Metabolism (MAPEGs) while the mitochondrial GSTs are also referred as kappa-class GSTs [32].

GSTs are able to conjugate GSH to a wide range of endogenous electrophiles and xenobiotics. The endogenous 4-hydroxynonenal (4HNE) is an aldehyde resulting from lipid peroxidation that forms covalent adducts with DNA and proteins; high concentrations of 4HNE have been associated with apoptosis, altered gene expression, neurodegenerative and cardiovascular diseases, cancer and metabolic syndrome [33,34,35]. GSTs are involved in the detoxification of 4HNE by conjugation with GSH; the resulting GS-HNE is actively excreted by efflux transmembrane transporters [35]. Similarly, aflatoxin B1, a toxic metabolite produced by fungi of the genus Aspergillus, is activated by cytochromes P450 into highly reactive epoxides with mutagenic properties; GSTs are involved in the conjugation of aflatoxin B1-exo-8,9 epoxide with GSH resulting in its elimination through the mercapturic pathway [36]. Acrolein is a highly reactive aldehyde resulting from burning tobacco, wood and plastic and when animal or vegetal fats and oils are overheated; acrolein is also a metabolite of the anticancer drug cyclophosphamide [37]. Acrolein forms adducts with DNA, proteins and lipids inducing cellular damage. GSTs are responsible for the first step in acrolein metabolization, consisting in its conjugation with GSH; after this first step, other reactions follow, leading to the urinary excretion of mercapturic acid metabolites [37,38]. GSTs are also involved in the reduction of organic hydroperoxides, such as phospholipids, DNA hydroperoxides and fatty acids, to their corresponding alcohols [30].

GSH directly participates in the neutralization of a toxic acetaminophen metabolite. Acetaminophen is metabolized primarily by sulfation and glucuronidation; however, cytochrome P450 enzymes convert 5–9% of acetaminophen to *N*-acetyl-p-benzo-quinonimine (NAPQI), which causes hepatotoxicity by binding to cellular macromolecules. NAPQI detoxification occurs primarily by GSH conjugation [39]. *N*-acetylcysteine treatment can prevent or limit liver injury by restoring hepatic GSH concentrations and it is used as an antidote in acetaminophen poisoning [40].

### 3.3. GSH in Protein Glutathionylation

*S*-gluta-thionylation is a reversible post-translational modification of a protein cysteine thiol group (P-SH) into a protein-S glutathione group (PSSG). Spontaneous PSSG formation depends heavily on the local GSH:GSSG ratio. The physiological GSH:GSSG ratio is not able to promote the formation of PSSG; only a dramatic GSH:GSSG ratio decline (i.e., from 100:1 to 1:1) would result in a 50% conversion of PSH into PSSG. Such conditions are extremely rare in vivo, therefore, in most cases, spontaneous PSSG formation is uncommon [41]. Differently, P-SOH groups, produced when P-SH groups are oxidized by ROS, are rapidly neutralized by GSH. Without the gluta-thionylation, P-SOH groups may be further oxidized resulting in protein deactivation [42]. Different radicals or even weak oxidants, such as NO, are also able to promote *S*-gluta-thionylation through the formation of an unstable intermediate that is reduced by GSH. In addition, P-SH and P-SOH can be modified by GSNO to form PSNO and/or PSSG [43].

### 3.4. Hepatocyte Cell Death and GSH: Switch between Necrosis and Apoptosis

**Figure 4:**
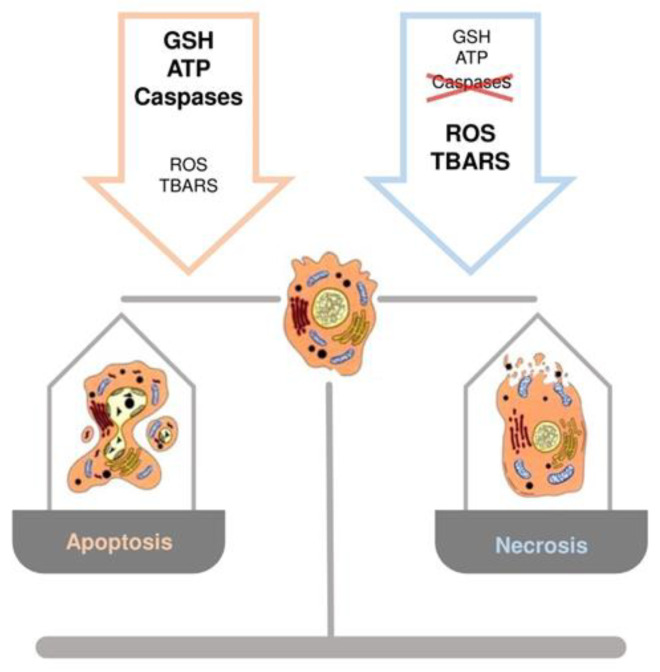
Schematic representation of the role of GSH, ATP, thio-barbituric acid reactive substances (TBARS), reactive oxygen species (ROS) and caspases in the regulation of cell death, apoptosis or necrosis.

The crucial role of GSH content in the regulation of hepatocyte cell death has been documented when an increase in ROS or a depletion of GSH occur [44,45]. A common observation, in experimental models of liver injury, is that low levels of ROS induce apoptosis, whereas higher levels lead to necrosis [9]. While apoptosis involves caspase activation, chromatin fragmentation and the phagocytosis of cell bodies, which minimizes inflammation, necrosis is defined by cell swelling and lysis with release of intracellular components, which elicits an inflammatory response [9]. Marked redox changes in hepatocytes, by using oxidants or GSH depletion agents, cause necrosis, whereas modest redox changes sensitize hepatocytes to tumor necrosis factor (TNF)-induced apoptosis by inhibiting the expression of the nuclear factor kappa-light-chain-enhancer of activated B cells (NF-kB)-dependent survival genes [44]. Other evidence obtained during rewarming of cold-stored rat hepatocytes, demonstrated that changes in GSH levels play a role in the mechanisms regulating apoptosis and necrosis [46]. The temporal occurrence of GSH depletion, as well as thio-barbituric acid reactive substances (TBARS) and ROS formation, and intracellular ATP content, represent the factors driving cells toward necrosis or apoptosis. A controlled amount of oxidative stress balanced by a sufficient amount of ATP will cause the apoptotic cascade to be activated. Differently, a marked depletion of GSH and ATP levels, accompanied by a profound increase in TBARS and ROS production, are associated with hepatocyte necrosis (Figure 4) [9].

The intracellular ATP concentration is a critical factor directing cells toward apoptosis or necrosis; in fact, apoptosis requires energy in the form of ATP to assemble the apoptotic machinery. On the contrary, during necrosis, ATP is dissipated due to the depletion of energy stores and a reduction of mitochondrial energy-transducing capacity [47]. Furthermore, apoptosis is known to be an energy consuming process because the cell requires an adequate ATP content for the correct function of both caspases and the apoptosome [45]. Oxidation of caspases by ROS has been proposed as one of the mechanisms involved in cell necrosis [45]. Caspases are cysteine proteases that are inactivated by the oxidation of thiol groups to disulfides, as well as other proteins involved in cell survival are, such as NF-kB or c-Jun N-terminal kinases (JNK) [44]. Under moderate ROS formation, apoptosis can start with the activation of pro-apoptotic factors, i.e., JNK, and inhibition of pro-survival factor, i.e., NF-kB, without changing the redox status of the caspases. Evidence suggests that the mitochondrial GSH pool plays a critical role for cell survival [48]. In ethanol sensitized hepatocytes, TNF-α increased the susceptibility of hepatocytes after mitochondrial GSH depletion; the restoration of mitochondrial GSH levels had protective effects against TNF-α [49].

Moreover, GSH plays a crucial role in the regulation of events leading to apoptosis or necrosis in several hepatic pathologies. In most cases, GSH represents a causative factor for disease progression. The different pathological settings will be described below.

**Figure 5:**
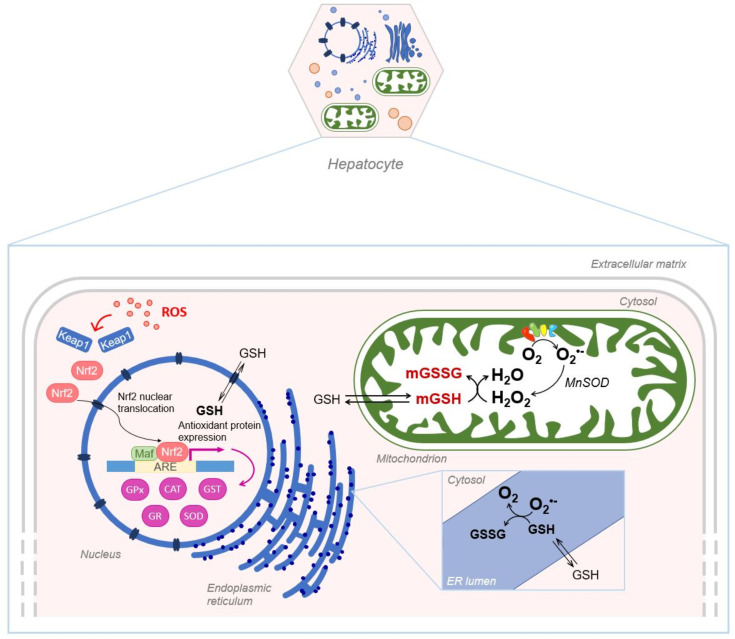
Schematic representation of GSH compartmentalization, in the nucleus, mitochondria and endoplasmic reticulum. mGSH: mitochondrial GSH; mGSSG: mitochondrial GSSG.

Despite its exclusive synthesis in the cytosol, GSH is distributed in intracellular organelles, including the nucleus, the mitochondria and the endoplasmic reticulum (ER) as depicted in Figure 5.

## 4. Liver and Intracellular Glutathione Compartmentalization

### 4.1. Nuclear Glutathione

The first hypothesis of a possible hepatic nuclear localization of GSH has been supported by the immunohistochemical localization of human GST2 not only in the cytoplasm of hepatocytes but also in the nucleus [50]. Later, the hepatic nuclear localization of GSH was also documented: high concentrations of this tripeptide have been found in the nucleus of intact hepatocytes and a nuclear/cytoplasmic concentration gradient was maintained by active mechanisms [51]. The nucleus does not partake in the synthesis of GSH because it lacks GCL and GS [52]. GSH transport from cytosol to nucleus is ATP dependent: studies in isolated nuclei obtained from rat liver demonstrated both GSH-stimulated ATP hydrolysis and ATP-stimulated GSH accumulation [53]. More recently, it has been shown that the GSH pool in the nuclear matrix derives from the cytoplasmic pool, probably via a nuclear pore-mediated translocation [52].

Alpha and Mu classes of GST2 superfamily enzymes, able to protect the cell from toxic endogenous or xenobiotic compounds, are abundantly expressed in rat liver, where they represent 43% and 56%, respectively, of the entire pool of cytosolic GSTs [54]. In rat hepatocytes, a significant fraction of dinitrosyl-diglutathionyl-iron complex is found in subcellular components, mainly in the nucleus, apparently entirely bound to Alpha class GSTs [55]. It was also reported that a considerable amount of GST, corresponding to 10% of the cytosolic pool, is electrostatically associated with the outer nuclear membrane, and a similar quantity is compartmentalized inside the nucleus [56]. Mainly, Alpha class GSTs, and in particular GSTA1-1, GSTA2-2, and GSTA3-3, are involved in this interaction, suggesting the existence of a protective enzyme barrier at the nuclear envelope, in which GSH plays an important role [56].

The expression of GPx and GR has also been demonstrated in nuclear fractions from rat hepatocytes [57]; the expression of these proteins in the nucleus underscores the key role of glutathione in the regulation of the cell cycle and in chromosomal organization; in fact, nuclear proteins, mainly histones and other chromatin-related proteins, have to be maintained in a reduced state for optimal function [52,57].

Combining information obtained from in vitro and in vivo studies, the key role of GSH in many nuclear processes in the liver has been further consolidated. Studies carry out in isolated nuclei obtained from the liver demonstrated GSH role in the modulation of the thiol/disulfide redox status of nuclear proteins and in the control of chromatin compacting and decondensation [53]. In vivo studies demonstrated the effect of GSH on gene expression through chromatin remodeling [58]. It has been also demonstrated that GSH content fluctuations in the liver are involved in many of the processes in which *c-myc* gene is overexpressed [44]. Torres et al. documented that, at hepatic physiological GSH content, the presence of deacetylase complexes and Id2 bound to the *c-myc* promoter led to a closed chromatin structure inaccessible to transcription factors [58]. On the contrary, when an acute GSH depletion occurs, Id2 and deacetylase complexes are released from the *c-myc* promoter, allowing transcription factors such as STAT3 to gain access to the *c-myc* promoter, leading to mRNA transcription [58].

Under specific circumstances such as in the active phases of cell proliferation, the nucleus accumulates GSH to much greater extent than the cytoplasm [59]. Brown et al. (2007) showed for the first time in an in vivo model that high hepatic GSH (1.5-fold), cysteine (15-fold), and glutamate cysteine ligase activity (1.5-fold) levels correlate with increased telomerase activity; on the contrary, telomerase activity was inhibited by GSH depletion with *N*-ethylmaleimide [60].

### 4.2. Mitochondrial Glutathione

Mitochondrial matrix is characterized by a high GSH concentration that plays an important role in defending mitochondria against oxidants and electrophiles. Mitochondrial GSH is particularly relevant in the disposal of hydrogen peroxide generated from superoxide anion by MnSOD, which otherwise may oxidize protein SH groups or peroxidize mitochondrial lipids [61].

GSH is negatively charged at physiological pH so it is unable to cross both the outer mitochondrial membrane (OMM) and the inner mitochondrial membrane (IMM). Studies on liver mitochondria have characterized GSH transport across mitochondrial membranes. Porins allow the diffusion of molecules such as GSH through the OMM [62]. As for the IMM, early findings in rat liver mitochondria suggested that active, energy-dependent processes inhibited by glutamate, carbonyl-cyanide p-(trifluoromethoxy)phenylhydrazone (FCCP) and ophthalmic acid were involved [63]. GSH is transported into hepatic mitochondrial matrix by a high-affinity transporter, which is saturated at a GSH concentration of 1–2 mM and stimulated by ATP. Another component with lower affinity is stimulated by ATP and ADP [63,64]. Other studies showed that the incubation of rat liver mitochondria with butyl-malonate (a dicarboxylate carrier inhibitor) and phenyl-succinate (a 2-oxoglutarate carrier inhibitor) inhibits GSH transport across IMM; thus, it has been possible to identify two anion carriers, members of the mitochondrial carrier family (SLC25): the dicarboxylate carrier (DIC; SLC25A10), and the 2-oxoglutarate carrier (OGC; SLC25A1) [48,65]. These two transporters are responsible for 45%–50% GSH uptake into the mitochondrial matrix in rat liver mitochondria, suggesting the involvement of other transporters [65]. Armeni et al. demonstrated the existence of an alternative GSH source in mitochondria: *S*-d-lactyl-glutathione (SLG), an intermediate of the glyoxalase system, is hydrolyzed by the mitochondrial glyoxalase II enzyme into D-lactate and GSH [66].

The mitochondrial transport of GSH is also dependent on membrane fluidity. For example, the 2-oxoglutarate carrier capacity changes with mitochondrial membrane fluidity: in rat liver mitochondria enriched in cholesterol, a decrease in mitochondrial GSH uptake was found [67]. On the other hand, higher mitochondrial membrane fluidity resulted in the recovery of GSH uptake rate [68].

Mitochondrial GSH is the primary defense against oxidative damage to mitochondrial membranes by ensuring the reduction of hydroperoxide groups and other lipid peroxides on phospholipids through the action of mitochondrial GSTs. Furthermore, GSH can also work non-enzymatically by reacting with electrophiles that are generated because of metabolic processes, although this reaction is greatly improved in presence of GSTs. Several cytosolic GST isoforms have been identified and the class Kappa isoform is the only GST present in mitochondria [69].

Under oxidative stress conditions, the increased levels of mitochondrial GSSG and protein disulfides are controlled by gluta-redoxin (Grx), a GSH-dependent disulfide oxidoreductase that catalyzes dithiol reactions, reducing GSH-protein mixed disulfides in a coupled system with GSSG-reductase and NADPH [70].

Regarding hepatic pathological setting, mitochondrial glutathione plays a critical role in the survival of hepatocytes during hypoxia through the regulation of mitochondrial generation of oxidative stress [71]. Furthermore, GSH depletion compromises mitochondrial function, sensitizes cells to toxicants worsening drug-induced hepatotoxicity [61].

Mitochondrial glutathione also participates to hepatic disease progression such as alcoholic liver diseases and non-alcoholic liver disease (NAFLD), as described in the below sections.

### 4.3. Endoplasmatic Reticulum Glutathione

The ER plays a crucial role in many cellular functions, such as synthesis of macromolecules, regulation of cellular calcium homeostasis, and maturation, processing, and transport of secretory and membrane-associated proteins [72]. Proteins assembled in the ER are rich in disulfide bonds that help to promote efficient folding and oligomerization of nascent polypeptide chains [73]. The activities of ER folding enzymes are highly dependent on the reduction/oxidation (redox) environment, and even small perturbations in the redox status greatly affect enzymatic activity and, as a result, protein folding kinetics [74]. It is widely believed that high concentration of GSH in the ER keeps protein disulfide isomerases (PDIs) in the reduced form [75]; these enzymes are the most important constituent of the oxidative machinery, together with the endoplasmic reticulum thiol oxidase (ERO1) [52]. The hepatic GSH transport in ER has been investigated and a bi-directional, saturable GSH transport was found in rat liver microsomal vesicles. GSH is preferentially transported in the reduced form as GSSG is virtually impermeable and is entrapped and accumulated in the microsomal lumen creating the oxidizing environment necessary for the formation of disulfide bonds and for the proper folding of proteins [76]. In any case, the GSH and GSSG movement and concentration into ER remain debated [77].

## 5. Glutathione and Liver Diseases

### 5.1. Non-Alcoholic Fatty Liver Disease

Non-alcoholic fatty liver disease (NAFLD) is a chronic and multifactorial disorder characterized by an excessive fatty acid (FA) accumulation in hepatocytes [78], due to an imbalance between FA uptake and disposal [79]. Noteworthy, NAFLD is the hepatic manifestation of the so-called “metabolic syndrome”, and is associated with a series of metabolic abnormalities, such as insulin resistance (IR), obesity, dyslipidaemia, hyperglycaemia, and hypertension. The pathology proceeds through different stages: from simple hepatic steatosis, it progresses toward non-alcoholic steatohepatitis (NASH), fibrosis, cirrhosis and in the most severe cases, toward hepatocellular carcinoma (HCC) [79]. According to the “multi-hit hypothesis”, several factors contribute to NAFLD development and progression: sedentary lifestyle and high fat diet can be related to obesity, dyslipidaemia and IR; changes in intestinal microbiome, genetic and epigenetic factors can also contribute [80]. NAFLD is estimated to affect 25% of the population worldwide [81] but, to date, there are no specific therapies available for the treatment [82], although some pharmacological strategies used for related disorder (e.g., diabetes) are also recommended for the treatment of NAFLD patients [79].

**Figure 6:**
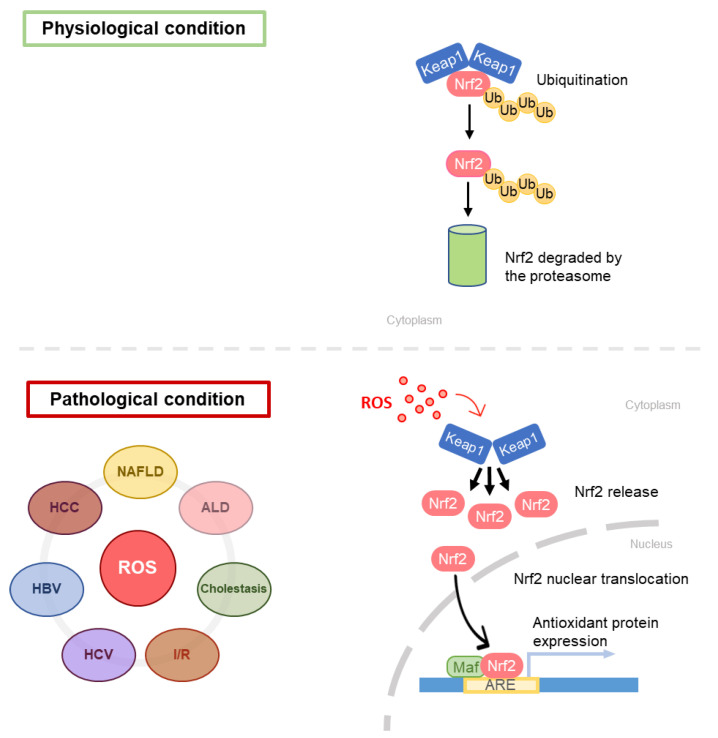
Schematic representation of Nrf2 pathway in different liver diseases. ARE, antioxidant response elements.

The main feature of NAFLD is the FA accumulation in liver parenchyma, due to an enhancement in FA uptake from plasma, in addition to an overstimulation of the de novo lipogenesis via SREBP-1c transcription factor activation [83]. The increase in FAs in liver parenchyma leads to an increased rate of *β*-oxidation, with the consequent electron outflow from the electron transport chain ETC [84]. The loss of electrons from ETC is associated with ROS overproduction and oxidative stress generation. Indeed, it has been widely demonstrated that oxidative stress also has a role in NAFLD pathogenesis [85]. Koruk et al. in 2004 showed that serum levels of malondialdehyde (MDA), GSH, and SOD were higher in NASH patients than in the control group, suggesting that the antioxidant system has a key role in NAFLD pathogenesis and disease progression [86]. Nrf2 seems to play a significant role in NAFLD development and oxidative stress response and is considered a novel potential target for the treatment of NAFLD. The role of Nrf2 in liver fibrosis has been clarified by Lu, who showed that GCL subunits and GS are regulated byNrf2, which binds AREs present in their promoters [2] (Figure 6). Furthermore, Zhang et al. have evidenced that an increase in the Nrf2 expression results in hepatic steatosis attenuation [21]. However, Nrf2 is not only implicated in the antioxidant response, but also plays a role in lipid metabolism [87,88]. Genes that are involved in lipid accumulation are negatively regulated by Nrf2 [87], and this finding supports the relevance of Nrf2 in NAFLD patients.

The role of ferroptosis has been evaluated as a determining factor for NASH onset [89]. Qi et al. showed that ferroptosis leads to a diminished hepatic expression of GPx4: experiments performed on MCD-fed mice revealed a reduced GPx4 expression after RSL-3 (a ferroptosis inducer) administration. Moreover, a higher amount of apoptosis inducing factors were found in mice liver, indicating that ferroptosis plays a key role in NASH-associated cell death [90]. On the other hand, the administration of a ferroptosis inhibitor, Liproxstatin-1, reduces hepatic lipid peroxidation and cell death [90].

GPx4 is highly expressed in liver mitochondria; Se, which has a role in GPx function, shows an antioxidant effect on liver; in fact, Se depletion is implied in the pathogenesis of hepatic inflammation and fibrosis [91], which are NAFLD-related injuries. In a case-control study with NASH patients, a positive correlation was found between the increase in Se levels and the GSH/GSSG ratio. This means that Se may have an important impact on GSH pathway, increasing the antioxidant capacity of the whole system [92]. It is worth noting that a diminished GSH level was observed in rats fed with a Se-deficient diet; specifically, an increase in n-6/n-3 fatty acid ratio has been shown that is one of the hits involved in NAFLD pathogenesis [93]. On the other hand, some authors have recently reported that high Se intake is associated with NAFLD progression [94]. Clearly, further exploration is needed to better elucidate the mechanisms at the basis of selenium role in the NAFLD establishment, considering its importance for GPx functioning.

*S*-adenosyl-l-methionine (SAMe) is a methyl donor involved in methionine metabolism. It is produced by the activity of methionine adenosyltransferase-1A (MAT1A), which is specifically present in the liver. It has been observed that MAT1A null mice present low GSH levels, combined with a reduced expression of lipid peroxidation genes. These mice develop steatohepatitis after eight months [95]. Furthermore, Montfort and colleagues showed in MAT1A^−/−^ mice a reduced SOD activity, possibly due to an increase in oxidative stress [96].

Cholesterol concentration in membranes is an essential factor for their fluidity and permeability. The mitochondrial GSH transporter 2-oxoglutarate carrier is involved in NASH development [97]. High amounts of free cholesterol in the liver reduce the activity of 2-oxoglutarate carrier. As a result, less GSH is translocated from the cytosol into mitochondria, favouring ROS accumulation and the consequent lipid peroxidation [98]. It has been demonstrated that the re-establishment of mitochondrial membrane fluidity at normal levels leads to the restoration of mitochondrial GSH levels [61]. Interestingly, ob/ob mice treated with atorvastatin did not show free cholesterol accumulation in mitochondria and the consequent mGSH reduction [98].

GSH involvement in NAFLD and NASH progression has been studied *in vitro*. In a nutritional model of steatosis in rats, Grattigliano et al. have evidenced an initial GSH increase, followed by a progressive depletion [99]. Also Yang et al. [100] noticed that fatty liver mitochondria from obese mice displayed enhanced GSH concentration. Probably, this result may be explained considering the increased GSH production in relation to a consistent increase in FAs in the liver. It has been also shown that in HepG2/C3A cells, the gene expression associated to GSH production is enhanced [101]. This response can be due to the rapid activation of the antioxidant system to protect the cell.

*In vitro* experiments evidenced that GPx was enhanced in steatotic immortalized human hepatocytes [102]; on the contrary, Videla et al. did not observe any difference in GPx expression between NAFLD patients and controls [103]. On the other hand, Videla and colleagues have demonstrated a robust correlation between NAFLD onset and GSH depletion in patients when compared with healthy individuals. Specifically, in NAFLD patients, they found an increase in protein oxidation/GSH ratio in comparison with controls in parallel with a significant decrease in SOD activity. In addition, low levels of CAT activity were observed in NASH patients, suggesting a reduced scavenging capacity of the antioxidant system. Changes in SOD and CAT activities in NAFLD and NASH patients are also accompanied by an increase in CYP2E1 activity [103]. CYP2E1 belongs to the cytochrome P450 family; it is involved in fatty acid oxidation [104] and is associated with NAFLD progression since it possesses an extremely high NADPH oxidase activity that leads to the production of ROS (H_2_O_2_, hydroxyl radical, and superoxide anion) [105]. Remarkably, the overexpression of CYP2E1 gene in an immortalized rat cell line results in GSH depletion and lipid peroxidation [106]. On the other hand, other authors reported an enhancement in GSH levels in HepG2 cells overexpressing CYP2E1 [107].

#### GSH in the Therapy of NAFLD

Through an open-label, single arm, multi-center, pilot study, Honda et al. proved a beneficial effect of oral GSH in NAFLD patients. They considered the reduction of ALT levels as the primary endpoint of the study since it is one of the principal NAFLD markers. The patients were treated for four months by oral GSH, and, after this period, blood ALT levels were considerably decreased, as well as the triglyceride content. Finally, the pilot study has evidenced that oral administration of GSH has positive effects on hepatic metabolism, improving NAFLD [108]. According to several studies, exogenous GSH seems to be degraded into its amino acidic constituents during digestive processes, which are used for the synthesis of endogenous GSH. Additional studies have clarified that the administration of the GSH precursors serine and glycine leads to an attenuation of NAFLD both in humans and in animal models [109,110]. The effect of glutamine administration on GSH levels has also been evaluated in a 5-fluorouracil-induced model of liver injury. In this model, endogenous glutamine production is not sufficient to counteract GSH depletion due to oxidative stress, and exogenous administration attenuates GSH depletion and MDA levels [111]. A more recent work investigated the supplementation of glutamine in combination with Western diet (WD), which resulted in a protective action against NASH development [112]. Rats fed with WD and glutamine showed less macro-vesicular fat accumulation, a decrease in lipid peroxidation and lower liver inflammation [112]. In another study on C57BL/6J mice fed with WD, liver morphology, blood enzymes and proinflammatory markers were improved in mice administered with Gln [113]. In a similar model, glutamine supplementation reduced oxidative stress in rats fed with a high-fat diet and induced an increase in GSH [114]. It is known that supplementation of serine and *N*-acetylcysteine (NAC) induces an increase in GSH levels. Indeed, NAC is a GSH precursor; it stimulates GSH synthesis, increases GST activity and promotes detoxification, scavenging ROS [103,115]. Its administration has been shown to restore GSH levels in NAFLD rats [116]. An experiment by Thong-Ngam et al. has shown that 20 mg/Kg NAC reduces oxidative stress in rats fed with high fat diet for 6 weeks to induce NASH development. In particular, serum GSH content was restored, and liver histology showed a normal morphological appearance in NAC-treated rats, with respect to controls [117]. An alternative pharmacological approach to counteract the oxidative stress related with NAFLD progression to NASH, consists in silymarin administration, which has been shown to restore GSH and GPx levels in serum [118]. It has also been proven that the supply of silymarin, a well-known compound with antioxidant properties extracted from the edible plant *Silybum marianum* [119,120], can help in terms not only of antioxidant effect, but also anti-inflammatory and antifibrotic effect [121]. It is worth noting that research into plant extracts to counteract oxidative stress are more and more common. Açai plant extract, for example, common name for *Enturpe oleracea*, contains significant amounts of polyphenols and anthocyanins that have shown an interesting antioxidant activity. De Freitas Carvalho et al. [122] investigated the contribution of açai extract in a murin model of NAFLD induced by a high-fat diet. They demonstrated an increase in GSH content, due to the augmented transcription of GCL. Moreover, açai extract administration led to a lower activity of GPx, SOD and CAT enzymes in the model used [122]. Another therapeutic plant extract that seems to have a beneficial effect on NAFLD-related oxidative stress is the blue honeysuckle (BH), common name for *Lonicera caerulea.* Kim et al. demonstrated that BHe (BH extract) administration led to an increase of the antioxidant defense system in high-fat diet NAFLD induced mice. In particular, in the groups treated with the BHe, the GSH content resulted increased, in a dose-dependent manner [123]. *Codonopsis lanceolata* plant extract were found to increase GSH/GSSG ratio [124], and, remarkably, Cunningham et al. proved that curcumin prevention led to a significant increase of GSH levels in female Wistar rats [125].

Not only plant extracts are useful to counteract oxidative damage in NAFLD condition, but also probiotics. For instance, treatments with 1, 2, 4 × 10^10^ cfu Jlus66 (strain of *Lactobacillus paracasei*) daily for 20 weeks in high-fat diet-fed rats ameliorated the SOD activity, as well as GPx activity [126].

### 5.2. Alcoholic Liver Disease (ALD)

**Figure 7:**
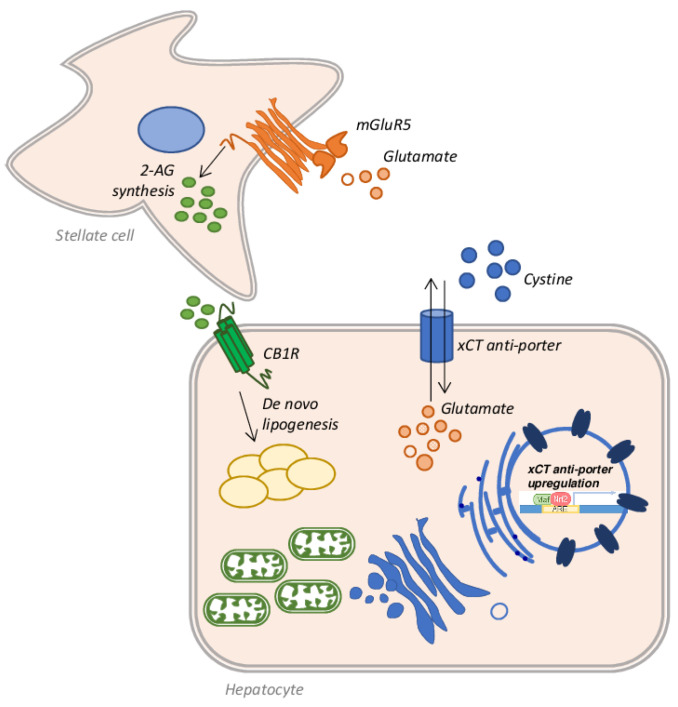
Schematic representation of the cross-talk between hepatocytes and stellate cells in alcoholic liver disease (ALD). Nrf2 activation leads to the upregulation of the xCT anti-porter system, which introduces cysteine within the hepatocytes and favors the efflux of glutamate in the extracellular space. Glutamate binds its mGluR5 receptor on the stellate cell surface, which synthetizes and releases 2-AG. 2-AG, once bound to its receptor CB1R, promotes the de novo lipogenesis mechanism activation and consequent lipid accumulation in the hepatic cells.

Alcoholic liver disease (ALD) is one of the commonest forms of liver disease globally. This is largely due to the ever-increasing consumption of alcoholic beverages, combined with a progressively sedentary lifestyle and wrong eating habits [127]. Under the spectrum of ALD a wide range of pathological conditions are addressed: simple steatosis, alcoholic steatohepatitis (ASH), fibrosis, cirrhosis, and HCC [128]. Among individuals who consume more than 60 g of alcohol per day and develop steatosis (about 90%), 25% progress to ASH and 10–20% of patients eventually develop cirrhosis [129]. ALD often fits into a picture already compromised by other factors such as hepatitis C virus (HCV) infection, or coexisting NAFLD, which accelerate the fibrotic process [130]. Several other factors can play an important role in the progression of ALD: environmental, genetic or behavioural causes can greatly influence the development of advanced stages of the disease [131]. Noteworthy, all these factors act by promoting an increase in oxidative stress via mitochondrial defects and the production of ROS and RNS [132]. Indeed, among the factors contributing to the progression of ALD to ASH and cirrhosis, oxidative stress certainly plays a key role [133]. Several works have shown that the accumulation of fat is toxic per se and causes lipo-toxicity [134]; the resulting fatty acid oxidation produces ROS, which can bind mitochondrial proteins, resulting in mitochondrial ROS production and mitochondrial dysfunction [135]. Moreover, it is well known that ethanol metabolism induces in the liver the production of ROS and RNS, altering mitochondrial structure and function and, concomitantly, decreasing many antioxidant mechanisms. This process, in turn, produces a dramatic decrease in ATP production, which alters the energy state of the cell, triggering the cell death process, in patients who chronically consume alcohol [136]. There are three main pathways employed by hepatocytes to metabolize ethanol and all produce ROS. The first pathway is catalyzed by alcohol dehydrogenase (ADH) and produces acetaldehyde from ethanol, leading to ROS and NADH generation [137]. The NADH produced can interfere with the mitochondrial electron transfer system producing ROS [138,139]. An additional amount of ROS is obtained by the microsomal ethanol-oxidizing system (MEOS) activity. This set of enzymes is located in the ER and the main player is the ethanol-inducible cytochrome P450 2E1 (CYP2E1), which converts ethanol to acetaldehyde, generating ROS [140,141]. Lastly, acetate is produced from ethanol in a reaction catalyzed by CAT in the peroxisomes [142]. Once acetaldehyde is obtained from ethanol metabolism, aldehyde dehydrogenase (ALDH) converts it into acetate, but if the system is overloaded, the excess of acetaldehyde reacts with mitochondrial GSH (mGSH), depleting the mitochondrial pool [143]. Since the mGSH acts in various molecular pathways involved in ROS detoxification, it is clear that the depletion of its stores by about 45–60% is strictly associated with the progression and development of ASH [144]. In in vivo rat models of ALD by intragastric ethanol feeding, it has been shown that chronic alcohol abuse causes a depletion of mGSH stores [145]. In chronic alcohol consumption, mGSH depletion is also caused by the disruption of GSH transport across the inner mitochondrial membrane, due to increased micro-viscosity caused by the accumulation of unesterified cholesterol on this site. A partial recovery in membrane fluidity of isolated mitochondria from ethanol-fed rat livers was obtained by the administration of SAMe, which restores the GSH mitochondrial pool [64,146]. A similar result was achieved employing taurourso-deoxycholic acid [147] or the fatty acid analogue 2-(2-methoxyethoxy)ethyl 8-(cis-2-n-octylcyclopropyl) (A2C), a fluidizing agent, in the same model of ethanol-fed rats [68]. Prolonged ethanol exposure, in fact, produces a reduction in the transport rate of GSH from cytosol to mitochondria, but not from mitochondria to cytosol, emptying the mitochondrial stores. The taurourso-deoxycholic acid administration restores GSH transport rate in hepatic mitochondria, replenishing the mGSH pool necessary to counteract the TNF-α mediated apoptosis [147]. The altered dynamic properties of the mitochondrial membrane, which affects GSH transport, could also be restored by A2C administration, as demonstrated in isolated mitochondria from long-term ethanol-fed rats [68]. Lastly, the depletion in the stores of mGSH may also originate from the activity of CYP2E1. CYP2E1 is located predominantly in the microsomal fraction of the cytosol, but is also expressed in mitochondria [148] and a three-fold increase in this enzyme was found in hepatic mitochondria isolated from ethanol-fed rats [149]. Not surprisingly, liver injury and oxidative stress are reduced in *Cyp2e1*^−/−^ ethanol-fed mice or when CYP2E1 is pharmacologically inhibited [150]. On the other hand, the oxidative stress was higher in transgenic mice overexpressing CYP2E1 [151], or in mice infected with an adenovirus to overexpress *Cyp2e1* gene and in HepG2 cells transduced with an adenovirus encoding the human *CYP2E1* gene [152]. An interesting finding by Cederbaum et al. showed that ROS produced by CYP2E1 induces an increase in Nrf2 expression, which promotes the upregulation of GSH-related genes as a compensatory mechanism [153]. Moreover, Nrf2 also upregulates the expression of the xCT anti-porter system that favors the further restoration of GSH stocks through the entry into hepatocytes of extracellular cystine and the efflux of intracellular glutamate [154]. In this context, the increased extracellular glutamate can bind to its receptor, the metabotropic glutamate receptor 5 (mGluR5) on the stellate cell surface, stimulating it to synthesize 2-arachidonoylglycerol (2-AG) [155], which in turn, once released into the extracellular space, binds to the cannabinoid receptor-1 (CB1R) on hepatocytes, triggering a mechanism of de novo lipogenesis and therefore of fat accumulation [156] (Figure 7). It is therefore clear that some components of this cross-talk between stellate cells and hepatocytes could be good pharmacological targets to be exploited in the treatment of steatosis. In fact, as demonstrated by Ferrigno et al., the selective blockade of the mGlu5 receptor attenuates fat accumulation in an *in vitro* model of mild steatosis [157].

#### Antioxidant Approach to the Treatment of ALD

At present, no pharmacological treatments for ALD and its progressive stages have been approved. The ideal treatment should be able to target all the issues of this pathology: increased inflammatory parameters, lipid accumulation, and oxidative stress generation. Obviously, given the enormous importance of oxidative stress in the progression of the disease, various treatments with antioxidant agents have been employed. As already mentioned, silymarin is a compound exhibiting antioxidant features [119,120]. In particular, silymarin shows antioxidant, anti-inflammatory, and anti-fibrotic properties useful in the treatment of ALD because it can reduce lipid peroxidation and increase GSH levels in cirrhotic patients after six months of treatment [158]. N-acetylcysteine (NAC), when used in animal models of early ALD, reduced lipid peroxidation, preventing hepatic GSH depletion [159,160]. However, these effects were not observed in severe stages of ALD, when used both alone or in combination with corticosteroids [161,162]. Among the emerging therapeutic strategies to reduce oxidative stress in ALD, several studies have shown the beneficial effect of folate, betaine, and metadoxine administration. Folate increases the conversion of homocysteine to methionine triggering the synthesis of SAM, useful to restore glutathione levels. Betaine acts as a methyl donor, providing a methyl group for the conversion of homocysteine to methionine. The synthetic drug metadoxine can restore NAD, ATP, glutathione, and adenosine levels both in the liver and brain, decreasing alcohol and acetaldehyde accumulation [163]. In a double-blind randomized trial, patients with ALD treated with 150 mg metadoxine for three months showed a considerable decrease in transaminase and γ-GT levels, as well as a reduction of steatosis, after the first month of treatment [164].

Several plant extracts have drawn attention for their antioxidant role in ALD. Quercetin, a compound belonging to the family of flavonoids, ameliorates lipid metabolism and ethanol-induced liver injury in animal models of ALD [165]. It has been demonstrated that it can induce antioxidant enzymes, increase GSH levels, reducing at the same time CYP2E1 activity and avoiding mitochondrial dysfunction and mGSH store depletion in rodents [166,167]. Similarly, honokiol, extracted from *Magnolia officinalis*, has been reported to improve ALD symptoms increasing hepatic levels of GSH and SOD activity in rats [168]; whereas barberine, a benzyl iso-quinoline alkaloid, increase levels of GSH and normalizes CYP2E1 hepatic expression in mice [169]. A similar replenishment of GSH stores was observed in primary rat hepatocytes exposed to ethanol and pretreated with curcumin [170].

It is well known that alcohol consumption affects the intestinal flora composition, so in recent years many groups have begun to pay attention on the role of probiotics in alcoholic liver disease. In particular, studies employing *Lactobacillus rhamnosus* GG in rats showed a reduction in alcohol-induced gut leakiness, oxidative stress and inflammation, both in the liver and intestine [171]. Moreover, *Lactobacillus rhamnosus* GG administration in ethanol-fed rats decreased both TNF-a and CYP2E1 protein and mRNA expression, enhancing Nrf2 protein expression [172].

### 5.3. Chronic Cholestatic Liver Injury

Cholestasis is a common impairment in bile formation, secretion, or flow. occurring in a plethora of human liver diseases [173] and is classified into two kinds: extrahepatic and intrahepatic. The first is the result of a mechanical blockade occurring in the biliary duct system caused by the presence of a gallstone, or a consequence of a pressure on bile ducts caused by tumor mass, bile duct tumors themselves, pancreatic tumors, pancreatitis, cysts, or primary sclerosing cholangitis. The intrahepatic cholestasis, instead, occurs inside the liver and could derive from ALD, amyloidosis, bacterial abscess, lymphoma, liver cancer, primary sclerosing cholangitis, viral hepatitis, or sepsis [174]. Moreover, cholestasis could be a metabolic impairment induced by genetic defects or scarring as seen in biliary atresia [175], or acquired as a side effect of many medications. In any case, the most commonly shared hallmark of different forms of cholestasis is an interruption of the enterohepatic circulation of bile acids and consequent retention of toxic bile salts in the bloodstream and at the intracellular level, leading to fibrosis and cirrhosis [173]. Toxic bile salts retention in the liver is accompanied by intracellular metabolic disorders, inflammation and reduced detoxification capacity [176], which may enhance the generation of oxidative stress and the accumulation of ROS and RNS in hepatocytes [177], leading to generalized systemic damage to major organs, starting from the liver and reaching the brain, heart, intestine, and kidney [178,179,180].

In the altered scenario of cholestasis, the retention of more hydrophobic bile salts, such as the glyco- and tauro-conjugates of deoxycholate, plays a major part in the onset of liver injury, causing hepatotoxicity due to mitochondrial dysfunction and production of ROS, with consequent oxidative damage [181,182]. Regarding GSH concentration during chronic cholestasis, conflicting data have been reported. However, many teams have demonstrated that a progressive decrease in GSH hepatocellular concentration occurs as the cholestasis progresses. Experiments conducted by Krahenbuhl et al. in a bile duct ligation (BDL) rat model of chronic cholestasis confirmed that the concentration of GSH and ubiquinone decreases during cholestasis, whereas an increase in lipid peroxidation is observed [183]. Moreover, GSH stores are consumed in animals subjected to experimental cholestasis or in hepatocytes exposed to toxic bile acids [184,185]. As shown by Neuschwander-tetri et al. and Serviddio et al., this fall in GSH stores is in part due to a decrease in GCL activity [184,186], a condition that, in rats subjected to the BDL procedure, can be prevented by the administration of urso-deoxycholic acid (UDCA) [186]. In fact, it has been demonstrated that UDCA can increase GCL expression in cultured rat hepatocytes [187]. Although the complete molecular mechanism underlying changes in GCL expression during chronic cholestasis or in response to UDCA has not yet been clarified, Yang and colleagues showed a transient and early increase in GSH-related enzymes, followed by their dramatic decrease in a second phase of cholestasis. In fact, they showed an early increase in both mRNA and protein expression of the two subunits of GCL (GCLC) and (GCLM), and also of GS from day 1 to day 7 after BDL, but these indices decrease below the baseline by day 14 to day 28 after BDL [188].

It is now well established that Nrf2, AP-1, NF-kB, and c-Myc are transcription factors able to induce the synthesis of GSH producing enzymes, both in humans and in rodents [189,190,191,192]. The mRNA and protein expression of these transcription factors remains unchanged after the BDL procedure, but GSH synthesis falls after 14 days of BDL. This occurrence is due to the impairment in Nrf2 ability to bind to AREs [188]. The binding of Nrf2 to AREs is controlled by the small Maf proteins (MafG, MafK, and MafF), which form heterodimers with Nrf2, effective in the activation of AREs, but at the same time, Maf proteins can homodimerize with each other, preventing the activation of AREs by Nrf2 [193,194]. Another protein able to block the Nrf2 activity is the Batch-1 protein. When an overexpression of Bach-1 occurs, as in high oxidative stress, it competes with Nrf2 to heterodimerize with small Maf proteins, repressing ARE-mediated gene expression [195]. Then, to understand why GSH synthesis decreases after BDL, it is necessary to know what happens to Maf and Bach-1 proteins. From day 14 to day 28, the hepatic nuclear content of small Maf proteins increases significantly. They form homodimers and prevent the bond between Nrf2 and AREs, which occurs early in the first period after the BDL procedure [188]. This fine regulatory mechanism at the level of gene transcription can therefore partly explain why the key enzymes in the synthesis of GSH decrease during cholestasis leading to an emptying of the GSH stores.

Another pathway involved in GSH depletion in cholestasis is strictly related to inflammation. In fact, in the compromised scenario of cholestasis, an inflammatory process is established, with a concomitant increase in levels of acute-phase proteins (APP) like serum TNF-α and biliary phospholipase A2, resulting in a further increase in oxidative stress highlighted by raised levels of MDA in the bile [176]. In rat models of extrahepatic cholestasis, it has been demonstrated that the regulation of some proteins of the acute phase depends on GSH, whose depletion causes beta-fibrinogen and haptoglobin up-regulation [196]. Lastly, another condition that affects GSH content is the bile regurgitation into the blood, caused by the intra-biliary pressure increase that causes the canalicular and Disse’s spaces able to establish a deep communication during prolonged obstructive cholestasis [197]. Thus, the toxic molecules can re-circulate between the bile and the hepatocytes, causing further depletion of GSH stores.

#### Antioxidant Approach for the Treatment of Cholestasis

Cholestasis affects not only the liver, but is a pathological condition that spreads in different organs. Unfortunately, currently no specific pharmacological strategy is optimal to treat cholestasis-induced organ injury. Nevertheless, several attempts have been made over the years to try to decrease the oxidative stress that plays a great part in its progression. Among them, UDCA has been demonstrated to be a valid candidate. In fact, it has been shown that UDCA pre-treatment protects cultured rat hepatocytes against oxidative stress induced by ROS, significantly increasing the total amount of GSH and thiol-containing proteins, via the up-regulation of GCL mRNA expression and metallothionein [187]. Serviddio et al. showed similar data on increased levels of GCL and γ-cystathionase mRNA in a BDL rat model [186]. Another compound that has been demonstrated to be useful in a rat model of extrahepatic cholestasis is silymarin. Indeed, silymarin-loaded gold nanoparticle, orally administered in BDL-rats for 7 days, improved liver function, reducing transaminase, γGT, and direct and total bilirubin serum levels, diminished oxidative stress markers both in serum and liver and, lastly, increased antioxidant support [198]. Agmatine has also shown antioxidant properties in a rat model of obstructive cholestasis. Agmatine is naturally produced in humans by arginine decarboxylation; it plays modulatory effects in various molecular pathways, including neurotransmitter systems, nitric oxide (NO) synthesis and polyamine metabolism [199]. Ommati et al. showed that the administration of agmatine in BDL rats for seven days after bile duct ligation was able to reduce ROS and nitric oxide formation and lipid peroxidation, and to increase mitochondrial GSH both in the liver and in the kidney in a dose-dependent way [200]. Moreover, the same research group recently obtained interesting data also on edaravone antioxidant capacities in the BDL rat model of obstructive cholestasis [201]. Edaravone showed potent antioxidant and radical scavenging activities in various pathologies, such as cerebral ischemia, stroke and amyotrophic lateral sclerosis [202,203,204], also improving oxidative stress-induced mitochondrial impairment in various diseases [205,206]. In their work, Ommati et al. demonstrated that BDL-rats treated with edaravone, at a dose of 1–10 mg/kg/day intraperitoneally for 14 days, show significant improvements in cholestasis-associated hepatic and renal injury, via reduction of ROS levels, lipid peroxidation, protein carbonylation, and GSSG levels. Antioxidant defenses, such as the total amount of GSH, and mitochondrial function were restored both in the liver and kidney [201]. Lastly, it is well known that prolonged cholestasis can induce cirrhosis and consequent disruption of gut barrier function and integrity [207]. This occurrence can favor bacterial lipopolysaccharide (LPS) translocation to the systemic circulation, leading to higher morbidity and mortality in cirrhotic patients [208]. In this case also, oxidative stress plays a notable role; in fact, NAC and dithiothreitol (DTT) induced a significant decrease in oxidative stress and restored intestinal antioxidant capacity in a BDL-induced model of cirrhosis in rats [209]. Finally, bombesin (BBS) and neurotensin (NT), two regulatory gut peptides, have been demonstrated to play a role in reducing hepatic oxidative stress in rats treated for 10 days with BBS and NT after BDL [210].

As regards plant extracts, quercetin also showed beneficial effects in the treatment of cholestasis. In fact, it has been shown in rats, that quercetin alleviates BDL-induced oxidative stress, leukocyte recruitment, NF-kB activation and pro-inflammatory cytokine secretion [211]. Moreover, another compound that showed antioxidant properties is hydroalcoholic extract of watercress, which is able to reduce oxidative stress in rats subjected to a BDL procedure, preventing hepatic protein oxidation and promoting GPx activity via antioxidant effect and free-radical scavenging [212].

### 5.4. Hepatic Ischemia/Reperfusion Injury

Hepatic Ischemia/Reperfusion (I/R) injury occurs during liver transplantation, resection surgery, and hypovolemic shock. The reperfusion occurring after ischemia elicits pathogenic processes, exacerbating injury caused by ischemia per se [213]. During ischemia, hepatocytes show morphological changes at the plasmatic membrane level, consisting in bleb formation. When blebs burst out, a release of enzymes and intracellular catabolites occurs, followed by the collapse of the ionic and electrochemical gradients, mitochondrial impairment, cellular acidosis, and Kupffer cells activation [214]. Instead, during the reperfusion period, reactive oxygen species, oxidative stress, mitochondrial dysfunction, and systemic inflammatory response take place [213]. After the I/R process, ROS are produced from different sources. Several protective mechanisms are triggered at the cellular level, many of them controlled by the Nrf2 transcription factor, which is normally inhibited by Keap1 [215,216]. When oxidative stress increases, Nrf2 moves to the nucleus enhancing the expression of antioxidant genes, such as NAD(P)H: quinone oxidoreductase 1 (NQO1), heme oxygenase-1 (HO-1), GCL, microsomal epoxide hydrolase, GST, and sulfiredoxin 1 [217]. GSH levels are controlled by Nrf2 via transcriptional activation of the GCL gene [189]. In a model of *Nrf2^−/−^* null mice subject to partial ischemia and reperfusion, the expression of several Nrf2-dependent genes was compromised, including GSTm1, NQO1, and GCL, resulting in the worsening of the redox state in comparison with livers from wild-type mice [218]. Moreover, a continuous release of GSH from hepatocytes to extracellular/vascular space detoxifies ROS generated by Kupffer cells, protecting the hepatic vasculature, as demonstrated in a in vivo model of partial no-flow ischemia/reperfusion injury [219].

#### Antioxidant Approach for the Treatment of Ischemia/Reperfusion Injury

Considering the relevant part played by oxidative stress in hepatic I/R injury, many attempts have been made to counteract this impaired condition. Different antioxidant agents showed clinical or experimental advantages if employed in I/R injury. For example, exogenous GSH administration in liver I/R at the dose of 100 μmol/h/kg showed significant beneficial effects both in cold and warm liver ischemia in rats [220,221]. Furthermore, in a model of isolated rat liver perfusion, GSH administration during cold preservation prevented hepatocyte damage, deterioration of hepatic circulation, and the fall of intracellular GSH levels during the reperfusion period [222]. Similar results were achieved by Schauer and colleagues, in a model of warm ischemia, followed by isolated rat liver perfusion [221]. In addition, many studies have also demonstrated the beneficial effects of NAC administration in hepatic ischemia, both in experimental and clinical settings. In *in vivo* and *ex vivo* models of hepatic ischemia and reperfusion injury [223,224], NAC administration had protective effects, increasing GSH levels and reducing ROS [225]. Moreover, when administered in a high dose, NAC is also able to support mitochondrial energy metabolism [226].

In a randomized Phase II study in patients undergoing liver transplantation, 30 mg/kg NAC administered 1 h before liver procurement and 150 mg/kg via portal vein in the recipient before liver implantation improved transplant survival by about 20% after one year and limited the risk of primary non-function. The authors argue that these findings should be kept in mind when suboptimal grafts are used, such as livers obtained from older people; moreover, early NAC systemic infusion is needed to reach a positive effect, filling the GSH stores before transplantation. All these beneficial effects of NAC administration go hand in hand with low cost and absence of adverse side effects [227]. Lastly, another attractive and innovative strategy in glutathione management is gene transfer. Considering that GSH has a very short half-life [228], gene transfer of glutathione synthesis components such as GCLC, GCLM, and GS could be a useful therapeutic approach in I/R injury through an increase in GSH intracellular concentration [215,216]. Gene therapy applied in hepatic I/R injury consists mainly in viral vectors, like recombinant virus transfection. In a rat model of I/R injury, the administration of high doses of mitochondrial SOD, via viral vector, inactivated NF-kB and activator protein-1, preventing I/R [229]. In addition, in a rat liver transplantation model using healthy and steatotic livers, the cytosolic and mitochondrial SOD delivered with adenovirus ameliorated the graft survival [230]. Lastly, in an in vitro study using murine NIH-3T3 cells, the transduction of human genes encoding for the peroxidase peroxiredoxin protected cells from oxidative stress [231]. Gene therapy, thus, could improve the effect of I/R and in particular could be applied in liver resection for tumors, or living donor liver transplantation. However, further studies are needed to clarify the effective efficacy of gene therapy in treating I/R injury [232].

Concerning plant derived compounds, *Hypericum perforatum* L. extract (HPE) showed antioxidant properties in studies focusing on I/R injury. In particular, rats subjected to I/R and pretreated with HPE displayed a significant decrease in hepatic damage markers such as ALT, AST, LDH and MDA. Moreover, a marked increase in CAT and GPx activity was found in livers from HPE-treated rats [233]. A similar effect was obtained also using rosmarinic acid, which showed anti-inflammatory and antioxidant role in rats subjected to ischemia and reperfusion procedure [234]. Intestinal microflora plays a role in many kinds of liver disease and also in I/R injury. It has been found that rats, treated with *Bifidobacterium Catenulatum* ZYB0401 and *Lactobacillus Fermentum* ZYL040, and then subjected to I/R, showed a decrease in TNF-a and hepatic MDA levels, accompanied by increased liver SOD activity [235].

### 5.5. Hepatitis C Virus (HCV) and Hepatitis B Virus (HBV)

**Figure 8:**
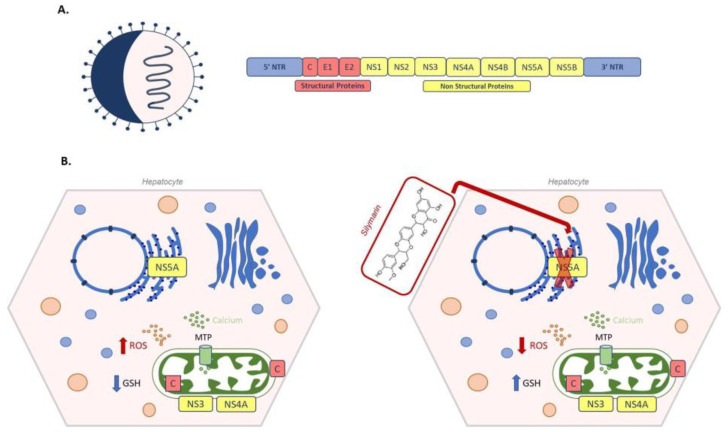
Schematic representation of hepatitis C virus (HCV) and silymarin treatment. (**A**) On the left a viral particle of HCV, on the right a HCV genome, with structural (Core, Envelope 1 and Envelope (2) and non-structural proteins (NS1, NS2, NS3, NS4A, NS4B, NS5A and NS5B). (**B**) On the left, an example of oxidative stress caused by viral proteins: core protein C binds to the inner and to the outer mitochondrial membrane; viral NS3/NS4 complex is associated with the outer mitochondrial membrane, while NS5A localizes on the endoplasmic reticulum. Viral NS5A increases calcium release from the ER into the cytoplasm. Through mitochondrial transition pore (MTP), calcium enters into the mitochondria, enhancing ROS production, together with C and NS3/NS4 proteins. On the right, silymarin administration blocks NS5A polymerase activity, ROS decreases and GSH levels increase.

According to the World Health Organization report, Hepatitis C virus infections worldwide total 71 million, with 400,000 deaths per year. After a six month period of acute infection, undiagnosed hepatitis C becomes chronic in about 80% of infected patients, leading to steatosis, fibrosis and cirrhosis; one patient out of five develop end-stage hepatocellular carcinoma [236,237]. Briefly, HCV is a positive single-stranded RNA virus belonging to the *Flaviviridae* family. Its genome is about 9600 bases in length and the translated polypeptide encodes for both structural (core, E1 and E2) and non-structural (NS1, NS2, NS3, NS4 A/B, NS5 A/B) proteins [238] (Figure 8A). Even though oxidative stress is considered a particular hallmark of hepatitis in both acute and chronic infection [239], contradictory data have been reported about the effect of HCV on intracellular GSH metabolism. In Huh7.5 cells infected by HCV genotype 2 an increase in GSSG and a decrease of GSH has been observed by Roe et al. [240], whereas de Mochel, using HCV-infected Huh7 cells, found an increase in GSH [241]. In the acute phase, viral replication takes place in the so called “membranous web” in the ER [242]. Moreover, ER stress induced by NS5A and core HCV proteins causes calcium release from the ER into the cytoplasm. Through mitochondrial transition pore (MTP), calcium enters into mitochondria, enhancing ROS production and misbalancing electron transport chain [243]. Oxidative stress can also be caused by the mitochondrial localization of viral proteins. Viral NS3/NS4 complex, associated with the outer mitochondrial membrane, cleaves the mitochondrial antiviral signalling proteins, abrogating the innate immune response pathway [244]. A ROS increase has also been associated to the presence of viral core proteins within the outer and inner mitochondrial membrane and in the mitochondria-associated membrane, which links the ER to mitochondria [243]. Korenaga et al. demonstrated that transgenic mice expressing HCV structural genes were characterized by an altered mitochondrial oxidative status. In transgenic mice, mitochondria GSH levels decreased compared to controls. Similarly, normal mitochondria treated with different concentrations of HCV core protein showed a significant oxidation of GSH. Moreover, the core protein increased calcium influx, and reduced electron transport chain complex I activity and consequent ROS production [245]. In line with these findings, hepatocytes overexpressing HCV core protein were also found to have decreased GSH levels and increased thioredoxin oxidation; while hepatocytes overexpressing viral NS5A protein have increased levels of GSH, CAT and SOD [246].

HCV also impairs redox state signalling pathways, such as Nrf2/ARE. It has been shown that, in the early phase of HCV infection, ROS-induced MAPK/JNK or PKC signalling mediates the activation of Nrf2 and Nrf2-activated antioxidant genes [243,247]. Other authors have reported an inhibitory effect on Nrf2 signalling due to the binding of sMaf to NS3 viral protein [248]. One of the side effects of Nrf2-downregulation is the inhibition of GPx4: in this case, lipid peroxides accumulate, worsening the damage and causing ferroptosis [249]. Conversely, NS5A viral protein is able to induce GPx4 by PI3K/AKT/mTOR pathway activation, protecting HCV infected cells against lipid peroxidation [250]. Another mechanism by which HCV causes oxidative stress is the induction of NADPH oxidases (NOX), which generate superoxide anions and hydrogen peroxide starting from molecular oxygen and NADPH [251]. In particular, during the acute phase, in which HCV replicates at a high rate, a marked oxidative stress due to the NOX4 isoform, ubiquitously expressed in parenchymal and non-parenchymal liver cells, has been observed, as well as a concomitant decrease in GSH. On the contrary, during the chronic phase, ROS production returns to basal levels and GSH content is restored [241]. In fact, in chronically HCV-infected patients, GSH content was found both in serum and hepatic tissue [252]. Furthermore, the HCV genotypes also affect glutathione levels in a different way. Patients affected by genotype 1b exhibited lower levels of hepatic and plasmatic glutathione, which correlates with increased lipoperoxidation markers as well as serum ferritin compared to patients with genotype 2a/2c and 3a and to controls [252]. Besides higher ferritin levels, probably due to activation of Nrf2-induced ferritin chains [253], patients can present higher iron content, which could lead to ferroptosis. Thus, the subtype 1a/1b, having not only low GSH but higher GSSG, cause more serious disease in contrast to the subtypes 2a/c, 3a and 4 [254]. GSH levels are re-established after antioxidant treatment (with a mixture of vitamin E, C, and zinc) alone or in combination with peg-interferon and ribavirin. Moreover, GPx and other antioxidant enzymatic idexesalso ameliorate after antioxidant supplementation. Concomitantly, peroxidation and iron content decrease, while zinc levels increase [255,256].

It is estimated that Hepatitis B virus (HBV) chronically infects 260 million people globally, causing almost one million annual deaths [257]. Besides chronic infection, HBV can cause acute, fulminant and occult infection [258]. HBV is a member of the *Hepadnaviridae* family. Its genome is a circular, partially double-stranded DNA, which encodes four overlapping open reading frames: C (core, HbcAg), X (HBx), P (DNA polymerase) and S (surface antigen, HbsAg). While C, P, S proteins have specific roles, HBx function is not completely understood: in fact, it seems to be implicated in signal transduction, DNA repair, transcriptional activation, as well as protein degradation inhibition. Moreover, it plays a central role in the induction of oxidative and inflammatory responses and in the progression to HCC [259].

The mechanisms of reactive oxygen species generation in HBV infections are several. ROS are useful for HBV assembly, by heat shock protein-90 (HSP-90) interaction with the core protein [260]. The presence of GSH, instead, changes HSP-90 conformation, abrogating HBV assembly [261]. HBV-induced ROS also enhance TNF-α, which activates JNK. In turn, JNK down-regulates both the expression and the activity of GPx in HBV-transgenic mice [262]. HBV-infected cell cultures and liver tissues have been demonstrated to induce antioxidant defence by the Nrf2/ARE pathway [263,264]. In particular, it has been shown that HBx protein forms a complex with p62 and Keap1; then, in HBV-infected HepG2.2.15 cells, the activated Nrf2 translocates to the nucleus [265] and stimulates ARE-regulated genes [264]. Moreover, HBV-infected HepG2.2.15 showed increased expression of GS, GR and GCL subunits when compared to non-infected HepG2 cells. Surprisingly, HepG2 cells exhibited higher levels of GSH with respect to infected HepG2.2.15, probably because GSH is engaged in facing HBV-induced ROS [266]. Short-term studies on HBV-inducible cell line HepAD38 also confirmed that HBV replication stimulates oxidative stress, as revealed by higher MDA levels after 24-h induction. Even though total GSH did not change during HBV infection, GSSG/GSH ratio has been observed to triple after 72 h HBV-induction, indicating that GSH depletion occurred in favour of its oxidized form [267]. However, in contrast with these findings, Nrf2/ARE downstream genes could be epigenetically suppressed. Hence, in HBV-hepatoma cells, DNA methyltransferase 3A (DNMT3A) hyper-methylates *NQO1* promoter, silencing it in a HBx-dependent manner [268]. Another example regards two isoforms of glutathione-S-transferase: M3 (GSTM3) and pi (GSTP1) [269]. Patients with chronic hepatitis B displayed a positive correlation between oxidative stress (MDA), methyltransferase expression (DNMT1 and DNMT3A) and methylation of GSTM3 and GSTP1. In HepG2.2.15, GSTP1 protein expression decreases, while, curiously, glutathione-S-transferase omega 1 (GSTO1) expression increases [270]. This could be traced back to the fact that GSTO1 is not under Nrf2/ARE control [271]: it acts more like glutaredoxins than GSTs [272] and is involved in the activation of inflammasome [273]. Similarly, in HBV mice transgenic for the antigen HbsAG, GSTP1 protein expression decreases, while Nrf2/ARE-independent glutathione-*S*-transferase kappa 1 (GSTK1) increases [274]. Furthermore, HBV infection is able to change the expression levels of antioxidant enzymes in an Nrf2-/ARE independent fashion. For example, HBx inhibited *GSTA2*, *GSTM1* and *GSTM2* gene expression by C/EBP-mediated mechanism [275]. Another way for HBx to induce oxidative stress is the suppression of proteins indirectly involved in the antioxidant defence, as observed in HBx-transfected HepG2 cells for the inhibition of selenoprotein P (SeP) [276] and in HBV-transgenic mice for selenium-binding protein 2 (Selenbp2) [274]. These data, in addition to low selenium content in HBV patients [277], exacerbate liver damage, hindering the activity of GPx, GST and thioredoxin reductase. However, as seen previously for HCV, the genotype affects the induced pathway. For example, genotype A presents a higher activation of Nrf2/ARE pathway than genotype G [263]. Similarly, in HepG2 cells infected by genotype D, GSTP1 is inhibited, but is not in genotypes A, B and C [278]. Even though much effort has been made to clarify the mechanisms at the basis of redox status changes in HBV in in vitro and in vivo models, the role of antioxidants has not been fully elucidated. However, several studies in plasma patients seem to agree on the increase in oxidative stress and on the decline of GSH and GSH-related enzymes [279]. In fact, increased MDA and transaminase levels in plasma of chronic HBV patients and diminished levels of GSH and GPx were reported, while GST content was upregulated [280,281]. In a study on Egyptian patients, along with MDA, GR also increases, whereas GSH, GPx and GST diminished [282]. GSH intravenous injections improved liver function, as indicated by reduced levels of transaminases and cytokines [283].

#### Antioxidant Approach for the Treatment of Viral Hepatitis C and B

Regarding viral hepatitis, several trials suggest that antioxidant therapy might be beneficial for patients with chronic infection. For instance, the supplementation of vitamins has been associated with the protection against free radical damage, HCV- or HBV-induced. The administration of GSH, a precursor or a derivative, inhibits the replication both in vitro and *in vivo*. The administration of a mixture of antioxidants, including GSH, decreased transaminase levels in comparison with placebo treatment and was well tolerated by chronic HVC patients [284]. *Nigella sativa*, both seeds and oil, has been demonstrated to have beneficial effects on HCV patients, thanks to its antioxidant properties [285]. In HCV patients the administration of sofosbuvir and ribavirin, along with black cumin seeds, derived from *N. sativa*, and ascorbate, reduced transaminase levels, increasing GSH and SOD, and decreasing GSSG, GGT, and MDA, when compared to sofosbuvir and ribavirin-treated patients [286]. Similar results were obtained for HBV treatment. In fact, an intravenous drip of 1200 mg of GSH was successful in decreasing the serum levels of transaminases, TNF-α, interleukin-6, and interleukin-8, compared with the control group, suggesting that GSH treatment can ameliorate liver function and suppress inflammation and hepatic fibrosis in chronic hepatitis B patients [283]. Even though a 48-h NAC treatment has been associated with about a 50-fold decrease in HBV assembly in HepG2-A5 cells [247], NAC has been revealed to be ineffective in acute viral B hepatitis treatment [287], However, other studies reported that NAC alone or in combination with other antiviral had no effects on plasma concentration of GSH, SOD and CAT for HBV [288,289] or HCV [290].

Silymarin treatment has been evaluated in viral hepatitis. Silymarin exerts its antioxidant effects in vitro not only by blocking NS5B polymerase activity, but mainly by inhibition of viral entry, stabilizing the membranes and yielding them less prone to fusion. Moreover, silymarin inhibits MTP activity and apoB secretion, limiting the viral spread [291] (Figure 8B). Similarly, silibinin, the major constituent of silymarin, has been demonstrated to inhibit HBV entry into hepatocytes in in vitro studies [292]. RT-PCR analysis revealed that the administration of silymarin upregulates genes involved in GSH metabolism, as well as in glutamate and citrate metabolisms, which indirectly could increase GSH [293]. Unfortunately, there are no univocal data about in vivo hepatoprotection mediated by silymarin [294]. In fact, the administration of silymarin formula MediHerb 600–1200 mg daily for 12 weeks did not change transaminase levels nor HCV RNA titer versus placebo [295]. Similar results were observed by Hawke [296] and Fried [297]. The supplementation of several antioxidants to 250 mg of silymarin demonstrated histological improvements as well as ameliorated ALT content [284]. As for HCV treatment with silymarin for HBV, there are also confusing reports. Wei et al., in their meta-analysis, concluded that silymarin seems to be safe and well tolerated by patients and, in combination with antiviral drug or protection liver drugs, serum transaminases were reduced [298].

Another strategy to counteract oxidative stress in viral hepatitis could be the use of probiotics. In fact, according to several studies, dysbiosis occurred, leading to a decrease in *Bifidobacteria* and *Lactobacilli* and the proliferation of *Bacteroidetes* and *Enterobacteriaceae* [299]. The lipopoli-saccharides of these Gram-negative bacteria causes endotoxemia in HCV and HBV patients [300], with the consequent decline in SOD and MDA levels [301] and increase in pro-inflammatory cytokines and ROS [302]. However, the administration of *Bifidobacteria*, as well as lactitol [301], or feces transplant [303], is associated with gut microbioma re-establishment [301].

### 5.6. Hepatocellular Carcinoma (HCC)

HCC represents about 75–85% of primary liver cancers cases, followed by intrahepatic cholangiocarcinoma (10–20%) and rare tumors, including hepatoblastoma [304]. HCC is a complex and heterogeneous tumor characterized by multiple genetic mutations in genes controlling cell proliferation, cell death, homeostasis and oxidative stress [305]. However, it is well known that the main risk factors for HCC development and progression are chronic infections due to viral hepatitis and alcoholic and non-alcoholic fatty liver disease, along with metabolic syndrome [306]. As reported in this review, since the liver diseases previously described have oxidative stress in common for their pathogenesis, it follows that the worsening of oxidative damage contributes to HCC development. Transient induction of Nrf2 has been observed to provide protection against oxidative stress. However, persistent activation of Nrf2, due to Keap1 or Nrf2 gene mutations, can drive HCC pathogenesis [307]. In line with these considerations, an Nrf2-dependent increase in GCLC expression levels and activity was observed in tumors, in comparison with peritumoral tissues [308]. Moreover, Nrf2 overexpression contributes to sorafenib-induced ferroptosis resistance both in in vitro and in in vivo models. In HCC cell lines, upon sorafenib administration, p62 inhibited Nrf2 degradation and enhanced its nuclear accumulation. On the contrary, C57BL/6 mice injected subcutaneously with *Nrf2* knockdown Hepa1–6 cells showed a decrease in tumor size and diminished NQO1 and HO-1 mRNA expression after sorafenib administration when compared to *Nrf2*-shRNA control mice [309]. In contrast, the administration of curcumin in HepG2 cells treated with the conditioned medium derived from hepatic stellate cells prevents HIF-1α stabilization, suppresses angiogenesis and upregulates GSH through the Nrf2 pathway [310]. Other contrasting data were reported on selenium dependent GPx. Se deficiency increases cancer incidence [311]. Hence, low Se levels have been observed in HCC patients’ blood, while its supplementation decreased the risk of cancer [311]. However, the seleno-protein GPx2 plays an important role in the progression of malignant tumors [312]. In HCC tissue, immunohistochemical analyses detected GPx2 overexpression when compared to adjacent non-tumor tissues [313]. Similarly, GPx4 and GPx7 were found to be overexpressed in HCC tissues and their levels correlate with the malignancy grade [314]. Several studies investigated GSH and GSH-related enzymes in HCC tissues. GSH content was lower in HCC tissues when compared to healthy livers, as well as selenium-dependent GPx, GST and GR [315,316]. Several studies report a decrease in GSTP1 in HCC tissues when compared to healthy or HBV patients [317], probably due to the hypermethylation of its promoter [318]. Likewise, the opposite has been shown. GSH content doubled in HCC biopsies as compared to normal liver, due to increase in GCL heavy subunit and GS. The presence of regulatory elements like ARE, activator protein-1 and NF-kB on GCL heavy subunits promoter enhances its transcription. Moreover, GSH increase has been positively correlated with HepG2 cell growth: inhibiting or increasing cellular GSH, DNA synthesis and cell proliferation were, respectively, reduced or improved [319]. Thus, GSH increase seems to promote tumour growth. In fact, GSH nuclear pool has also been reported to be fundamental for the advancement of cell cycle from G1 to S-phase in healthy tissues. A shift toward GSSG redox state, instead, keeps the cell cycle blocked into G1 phase [320]. Recently, Cheng et al. confirmed the presence of increased GSH content in HCC tissue, when compared to neighbouring healthy tissues. Interestingly, they found a difference in oxidative markers in plasma before and after the tumor resection: after surgical intervention, MDA concentration lowered and GSH levels were restored [321]. Besides the nuclear GSH pool, in HCC cells and human HCC tumors, mitochondrial GSH increase has also been observed [322]. Indeed, the 2-oxoglutarate carrier is over-expressed in HCC, ensuring unlimited mGSH levels, useful to counteract ROS. Silencing this carrier, instead, is associated with decreased liver tumorigenesis *in vivo*.

#### Antioxidant Approach for the Treatment of Hepatocellular Carcinoma

Contrasting data 0n the efficacy of GSH in HCC have been reported. GSH and antioxidant agents are often recommended in hepatic chronic diseases to prevent hepatocellular carcinoma [121,323]. In preclinical studies, silymarin treatment has been associated with ROS decrease in HCC cell culture [324], and in HepG2 cells it decreased cellular proliferation [325]. Similarly, in N-nitroso-diethylamine-induced rat hepatocarcinogenesis, silymarin induced a decrease in lipid peroxidation concomitantly to an increase in GSH [325,326]. The administration of quercetin, a plant polyphenol, was associated with enhanced GSH levels and a reduction in lipid peroxidation, resulting in the inhibition of cancer development, both in vitro and in vivo models [327,328]. Tea polyphenols seem to have positive effects on gut microbioma, repressing pathogenic bacteria in favour of friendly ones. The same role is exerted also by pro-anthocyanidins, found for example in almonds, grapes and chocolate [329]. Phenols contained in curcumin were reported to be able to arrest cell cycle and inhibit proliferation and metastasis [330].

However, it is also well known that the administration of antioxidants such as GSH or its precursors, as well as vitamins, increases chemoresistance [331]. In fact, elevated GSH and GCLC levels were found in different malignant tumors, masking the tissue more resistant to chemotherapeutic drugs such as cisplatin [332,333]. GST isoforms have also been found to increase HCC cells’ resistance to chemotherapy [334]. For this reason, GSH depletion has been considered a potential strategy to sensitize tumor cells to therapy [331]. Tompkins et al. demonstrated that the disruption of mitochondrial pyruvate carrier downregulates GSH metabolism, with lethal effects on carcinoma-induced hepatocytes [335]. The generation of oxidative stress could also be used as a therapy. Indeed, three hepatocellular cancer cell lines (Huh-7, HepG2 and PLC-PRF-5), treated with the anticancer sorafenib in combination with the anti-inflammatory diclofenac, showed a greater oxidative stress and cell death, associated with GSH decrease [336]. However, it has been shown that the use of amifostine, an antioxidant scavenger, could exert a protective effect on normal tissues [337]. In fact, because its thiol is susceptible to pH and alkaline phosphatase, it acts only in normal tissues, without affecting the anticancer effects of chemotherapy and ionizing radiation [338,339,340].

## 6. Conclusions

GSH represents one of the most commonly investigated redox-active molecules, as changes in its content contribute to the pathogenesis of many diseases. It is now evident that GSH is not merely a ROS and RNS scavenger. In fact, GSH has additional roles in cell physiology, such the metabolization of toxic substances, cell signaling through protein S-glutathionylation and cell survival. Oxidative stress contributes to the pathogenesis of many liver diseases: it is involved in SEC activation and fibrotic progression in NAFL and ALD; it induces mitochondrial dysfunction in cholestasis and in ischemia/reperfusion injury; it is also a hallmark of viral hepatitis and hepatocellular carcinoma. Consequently, the supplementation of GSH or GSH precursors has been demonstrated to be a suitable contribution to the therapy for many hepatic conditions.

## Abbreviations

2-AG	2-arachidonoylglycerol
4-HNE	4-hydroxynonenal
ADH	Alcohol dehydrogenase
Akt	Protein kinase B
ALD	Alcoholic liver disease
ALDH	Aldehyde dehydrogenase
ALT	Alanine aminotransferase
AP-1	Activator protein 1
APP	Acute phase protein
ARE	Antioxidant response element
ASH	Alcoholic steatohepatitis
ATP	Adenosine triphosphate
BBS	Bombesin
BDL	Bile duct ligation
Bhe	Blue honeysuckle extract
CAT	Catalase
CB1R	Cannabinoid receptor-1
CHB	Chronic hepatitis B
CYP2E1	Cytochrome P450 2E1
DIC	Dicarboxylate carrier
DILI	Drug-induced liver injury
DNMT	DNA methyltransferase
DTT	Dithiothreitol
ER	Endoplasmic reticulum
ETC	Electron transport chain
FA	Fatty acid
FCCP	carbonylcyanide p-(trifluoromethoxy)phenylhydrazone
GCL	l-glutamate l-cysteine γ-ligase
GCLC	GCL catalytic monomer
GCLM	GCL modifier monomer
GPx	Glutathione peroxide
GR	Glutathione reductase
Grx	Glutaredoxin
GS	GSH synthetase
GSH	Reduced glutathione
GSSH	Oxidized glutathione
GST	Glutathione S transferase
HBV	Hepatitis B virus
HCC	Hepatocellular carcinoma
HCV	Hepatitis C virus
HIF-1α	Hypoxia-inducible factor 1 alpha
HO-1	Heme oxygenase-1
HPE	*Hypericum perforatum* L.
HSP90	Heat shock protein 90
I/R	Ischemia/reperfusion
IL-6	Interleukin-6
IMM	Inner mitochondrial membrane
IR	Insulin resistance
JNK	c-Jun N-terminal kinases
Keap1	Kelch-like ECH-associated protein 1
LPS	Lipopolysaccharide
MAF	Musculoaponeurotic fibrosarcoma oncogene homolog (avian)
MAPK	Mitogen-activated protein kinase
MAT1A	Methionine adenosyltransferase-1
*Mcp1*	Monocyte chemoattractant protein 1
MDA	Malondialdehyde
MEOS	Microsomal ethanol-oxidizing system
mGSH	Mitochondrial glutathione
MnSOD	Manganese-dependent superoxide dismutase
mTOR	Mammalian target of rapamycin
MTP	Microsomal triglyceride transfer protein
NAC	*N*-acetylcysteine
NADPH	Nicotinamide adenine dinucleotide phosphate
NAFLD	Non-alcoholic fatty liver disease
NAPQI	*N*-acetyl-p-bemzoquinone imine
NASH	Non-alcoholic steatohepatitis
NF-kB	Nuclear factor kappa-light-chain-enhancer of activated B cells
NO	Nitric oxide
NOX	NADPH oxidase
NQO1	NAD(P)H:quinone oxidoreductase 1
Nrf2	Nuclear factor erythroid 2-related factor 2
NT	Neurotensin
OGC	2-oxoglutarate carrier
OMM	Outer mitochondrial membrane
PAI1	Plasminogen activator inhibitor 1
PDI	Disulfide isomerase protein family
PI3K	Phosphoinositide 3-kinase
RNS	Reactive nitrogen species
ROS	Reactive oxygen species
SAM	*S*-adenosyl-l-methionine
SLG	*S*-d-lactoylglutathione
SOD	Superoxide dismutase
SREBP-1c	Sterol regulatory element-binding protein 1C
TBARS	Thiobarbituric acid reactive substances
TNF-α	Tumor necrosis factor alpha
UDCA	Ursodeoxycholic acid
WD	Western diet
xCT	Cystine/glutamate antiporter
γ-GT	Gamma-glutamyltransferase
